# Lateral Flow Immunoassay for Rapid Detection of Grapevine Leafroll-Associated Virus

**DOI:** 10.3390/bios8040111

**Published:** 2018-11-15

**Authors:** Nadezhda A. Byzova, Svetlana V. Vinogradova, Elena V. Porotikova, Uliana D. Terekhova, Anatoly V. Zherdev, Boris B. Dzantiev

**Affiliations:** 1 A.N. Bach Institute of Biochemistry, Research Center of Biotechnology of the Russian Academy of Sciences, Leninsky Prospect 33, Moscow 119071, Russia; nbyzova@inbi.ras.ru (N.A.B.); zherdev@inbi.ras.ru (A.V.Z.); 2 Institute of Bioengineering, Research Center of Biotechnology of the Russian Academy of Sciences, Leninsky Prospect 33, Moscow 119071, Russia; svetlana.vinogradova@biengi.ac.ru (S.V.V.); plantvirus@mail.ru (E.V.P.); terekhova_uliana@mail.ru (U.D.T.)

**Keywords:** immunochromatography, gold nanoparticles, test strips, phytopathogens, on-site testing, agricultural control

## Abstract

Grapevine leafroll-associated virus 3 (GLRaV-3) is one of the main pathogens of grapes, causing a significant loss in yield and decrease in quality for this agricultural plant. For efficient widespread control of this infection, rapid and simple analytical techniques of on-site testing are requested as a complementary addition for the currently applied hybridization (PCR) and immunoenzyme (ELISA) approaches. The given paper presents development and approbation of the immunochromatographic assay (ICA) for rapid detection of GLRaV-3. The ICA realizes a sandwich immunoassay format with the obtaining complexes ((antibody immobilized on immunochromatographic membrane)–(virus in the sample)–(antibody immobilized on gold nanoparticles (GNP)) during sample flow along the membrane compounds of the test strip. Three preparations of GNPs were compared for detection of GLRaV-3 at different dilutions of virus-containing sample. The GNPs with maximal average diameters of 51.0 ± 7.9 nm provide GLRaV-3 detection for its maximal dilutions, being 4 times more than when using GNPs with a diameter of 28.3 ± 3.3 nm, and 8 times more than when using GNPs with a diameter of 18.5 ± 3.3 nm. Test strips have been manufactured using the largest GNPs conjugated with anti-GLRaV-3 antibodies at a ratio of 1070:1. When testing samples containing other grape wine viruses, the test strips have not demonstrated staining in the test zone, which confirms the ICA specificity. The approbation of the manufactured test strips indicated that when using ELISA as a reference method, the developed ICA is characterized by a sensitivity of 100% and a specificity of 92%. If PCR is considered as a reference method, then the sensitivity of ICA is 93% and the specificity is 92%. The proposed ICA can be implemented in one stage without the use of any additional reactants or devices. The testing results can be obtained in 10 min and detected visually. It provides significant improvement in GLRaV-3 detection, and the presented approach can be transferred for the development of test systems for other grape wine pathogens.

## 1. Introduction

Viral diseases are one of the main threats to the cultivation of grapes. More than 60 viruses are pathogenic for grapes [1,2,3]. Additional agricultural risks result from grapes being a vegetatively propagated perennial crop in which pathogens accumulate while the plants grow in vineyards. Grapevine leafroll disease (GLD) is considered as one of the most common and economically significant viral diseases of this plant, which is caused by viruses of the *Closteroviridae* family [4,5]. Grapevine leafroll-associated virus 3 (GLRaV-3), a member of the genus *Ampelovirus*, is the infectious agent associated with GLD. Particles of GLRaV-3 virus are filamentous structures with a length of 1800 nm and a diameter of 12 nm with a spiral arrangement of repetitive protein subunits [4].

The infection spreads via planting materials during plant grafting as well as through insect vectors [6,7,8]. In plants infected with the virus, growth slows down, leaf area decreases, and shoots become weak. A low content of chlorophyll, manifested as a change in leaf color, leads to a decrease in photosynthetic activity. As a result, the sugar content in the berries is reduced by 30%, the berries become smaller, and their ripening time increases. The complex of pathological changes caused by the virus leads to a decrease in the commercial value of table grapes [6,8,9]. The yield decreases when plants are infected with GLRaV viruses—depending on grape variety, rootstock, the presence of a mixed infection, and climatic and soil conditions—ranges from 15% to 40% [4,10,11,12]. In connection with the negative effects described above, the grapevine leafroll-associated viruses are included by the European and Mediterranean Plant Protection Organization (EPPO) on the list of controlled grape pathogens [13,14].

Traditional diagnostics of phytopathologies are based on a visual assessment of symptoms. However, viral diseases of grapes vary significantly in symptoms and may also be asymptomatic [10,15,16]. Effective control of infections is possible only with the use of molecular and immunochemical methods [17,18]. In modern practice, the use of the polymerase chain reaction (PCR) [19,20,21], enzyme-linked immunosorbent assay (ELISA) [22,23,24,25] and various biosensors and some portable tests [26,27,28] for these purposes dominates. However, for the effective protection of plants, these methods, which require significant expenditures on reagents and equipment and can only be implemented in laboratory conditions, are not enough. To ensure mass primary screening, the methods used should include rapid, simple testing which can be carried out in non-laboratory conditions.

Today, immunochromatographic assays (ICAs) satisfy these requirements to the greatest extent. ICA is performed using test strips, multimembrane composites onto which specific reagents have been pre-applied. Contact of the test strip with a sample initiates movement along the membranes of its liquid components and immunoreagents, including a colored marker conjugated with antibodies. After this movement (in 10–15 min), specific immune complexes are formed on different parts of the test strip, which can be simply identified by the color of the linked label [29]. The above-described features of ICA allow for the testing of non-laboratory conditions in a short time with minimal sample preparation, without the use of additional reagents and devices, and with a simple detection and interpretation of the obtained results [30,31].

Although ICA is being actively developed and used to diagnose viral and bacterial pathogens of various economically significant plants [27,30], there are no descriptions in the literature on the development of ICA for grape viruses. The only two publications report the use of commercial test strips [32,33], which are currently not represented in manufacturers’ catalogs.

In this regard, the purpose of the presented work was the development and approbation of an ICA for the detection of GLRaV-3. An important additional task solved in the framework of this study was the determination of the requirements for the marker used in the test system, namely gold nanoparticles (GNPs). GNPs are the most common marker in ICA. Preparations of GNPs of various sizes can be obtained by varying the conditions of synthesis [34]. However, the question of the optimal size of GNPs for ICA remains open. There are empirical recommendations for using GNPs in ICA with an average diameter of 30–40 nm, but the characteristics of the compound being determined and the format of the analysis can significantly influence this choice [35,36,37]. Therefore, using GNPs of different diameters, this study includes a comparison of the analytical characteristics of the test system.

## 2. Results

**Figure 1:**
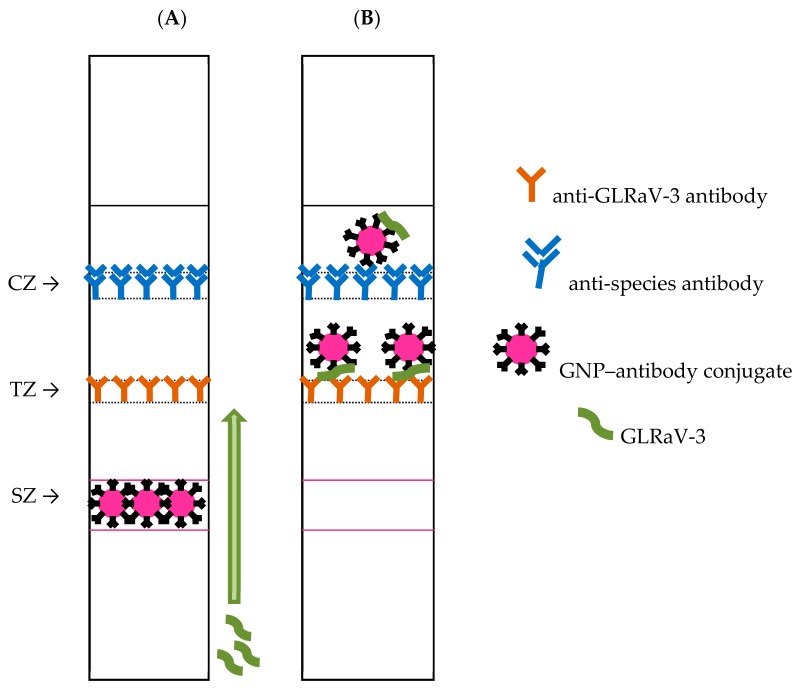
Scheme of the test strip for sandwich immunochromatographic assay (ICA) of GLRaV-3 before (**A**) and after (**B**) analysis.

The sandwich ICA with the formation of (immobilized antibody)–(antigen)–(GNP-labeled antibody) complexes has been chosen for realization in this study. It is known to provide a lower detection limit than other types of immunoassays [38]. The implementation of the sandwich immunoassay for the test strip is presented in Figure 1. An anti-species antibody was used to form the control zone (CZ), and an anti-GLRaV-3 antibody was used to form the test zone (TZ). GNPs–antibody conjugate applied to the start zone (SZ) of the test strip. The antigen-containing sample is adsorbed by the absorbing part of the test strip. The antigen reacts with the GNPs–antibody conjugate, forming a double antigen–antibody complex. This complex with the fluid flow reaches TZ and interacts with adsorbed specific antibodies. Ternary antibody–antigen–antibody complex is formed by this way. An excess of the antigen–antibody complex reaches CZ and forms a colored complex with anti-species antibody there.

### 2.1. Synthesis and Characterization of Reagents for ICA

**Figure 2:**
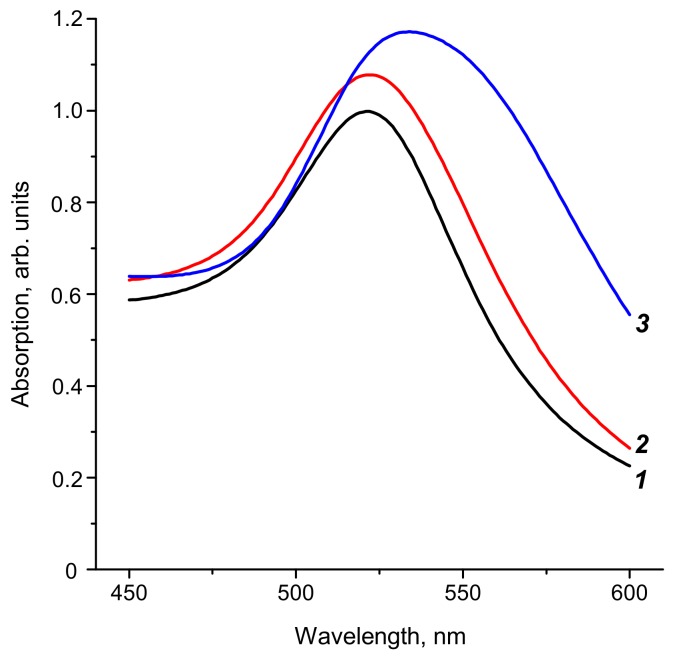
Absorption spectra of gold nanoparticles (GNPs). The mean sizes are 18.5 ± 3.3 nm (1), 28.3 ± 3.3 nm (2), and 51.0 ± 7.9 nm (3).

Three preparations of GNPs were synthesized. Their absorption spectra (Figure 2) are typical for colloidal non-aggregated gold. Some shift of the spectral maximum into the long wave region is observed with increasing particle size. The size and uniformity of GNPs were studied using transmission electron microscopy. As follows from Figure S1, the form of GNPs in all preparations was close to spherical, and the preparations did not contain aggregates. The obtained photographs were used to calculate the diameter of the particles; 93 to 153 measurements were used for the calculations. The mean values of GNPs diameters and mean deviations of these parameters were found to be 18.5 ± 3.3 nm (Figure S1B), 28.3 ± 3.3 nm (Figure S1D), and 51.0 ± 7.9 nm (Figure S1F).

**Figure 3:**
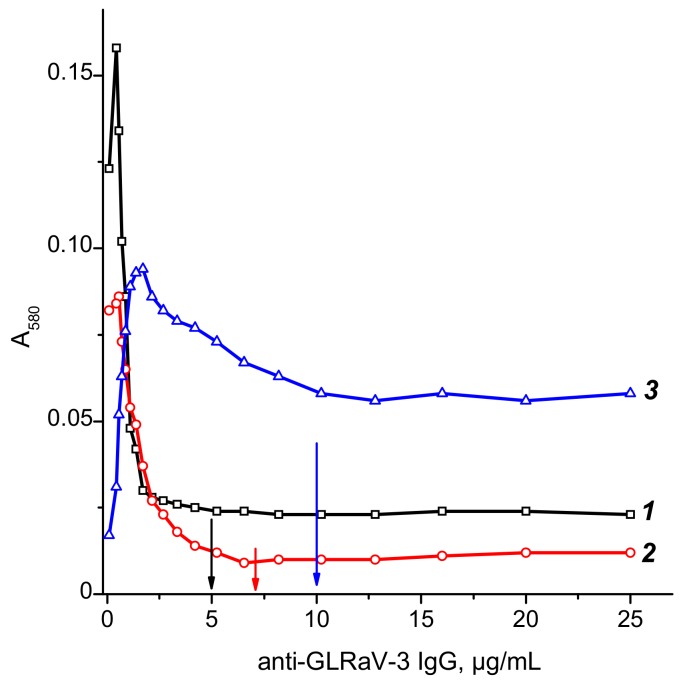
Flocculation curves of antibodies’ immobilization on the surface of GNPs with diameters of 18.5 ± 3.3 nm (1), 28.3 ± 3.3 nm (2), and 51.0 ± 7.9 nm (3). Arrows indicate the antibodies’ concentrations for the stabilization of the GNP’s surface.

To determine optimal antibody concentrations for conjugation with GNPs, flocculation curves were used as recommended by Hermanson [39]. These curves, measured at A_580_ (Figure 3), reflect the aggregation of the conjugates at high ionic strength in the presence of 10% sodium chloride. As can be seen, the addition of antibodies initially leads to an increase in absorption, after which the A_580_ value decreases and reaches a plateau. The curves part with unchanged A_580_ and, correspondingly, without aggregation, are considered as the conditions with complete stabilization of GNP surface by immobilized antibodies. Specifically, these concentrations were equal to 5 μg/mL, 7 μg/mL, and 10 μg/mL for the GNPs, with mean diameters of 18.5 nm, 28.3 nm, and 51.0 nm, respectively.

For these conditions of synthesis, the molar (antibody: GNPs) ratio during the conjugates’ synthesis was, respectively, 50:1, 125:1, and 1070:1. Note that monolayer immobilization of antibodies requires antibody: GNPs ratios, which are GNPs 54:1, 125:1, and 420:1, respectively, for these diameters of GNPs. The average contact area of an antibody molecule with a GNPs surface was 20 nm^2^ [40]. From a comparison of these ratios, it can be seen that the quantities of antibodies used in the synthesis either correspond or exceed (in the case of the largest GNPs) the minimum requirements for the formation of a monolayer.

The antibodies to GLRaV-3 used in this work are a mixture of polyclonal (PAb) rabbit and monoclonal (MAb) mouse antibodies, which differ from each other in the structural properties of the molecules and, potentially, in their ability to absorb on the GNP surface. The reason to work with this preparation is its (stated by manufacturer) efficiency in recognition of a broad spectrum of GLRaV-3 variants. The combination of PAb and MAb against different purified viruses and recombinant coat protein provides binding with all described isolates of GLRaV-3, including genetic variant groups I, II, III, VI, NZ1, NZ2, and NZ1-VM [41].

**Figure 4:**
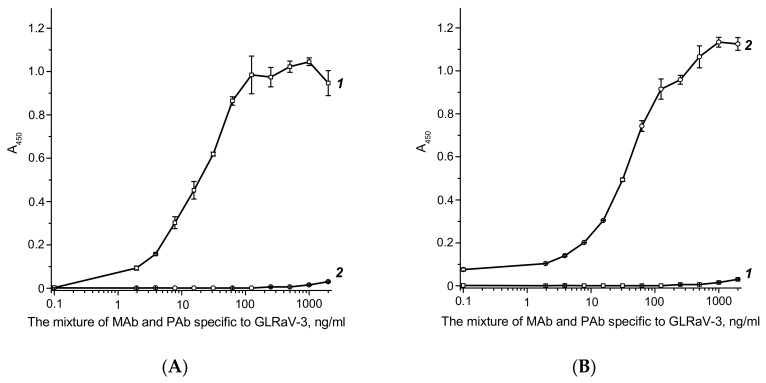

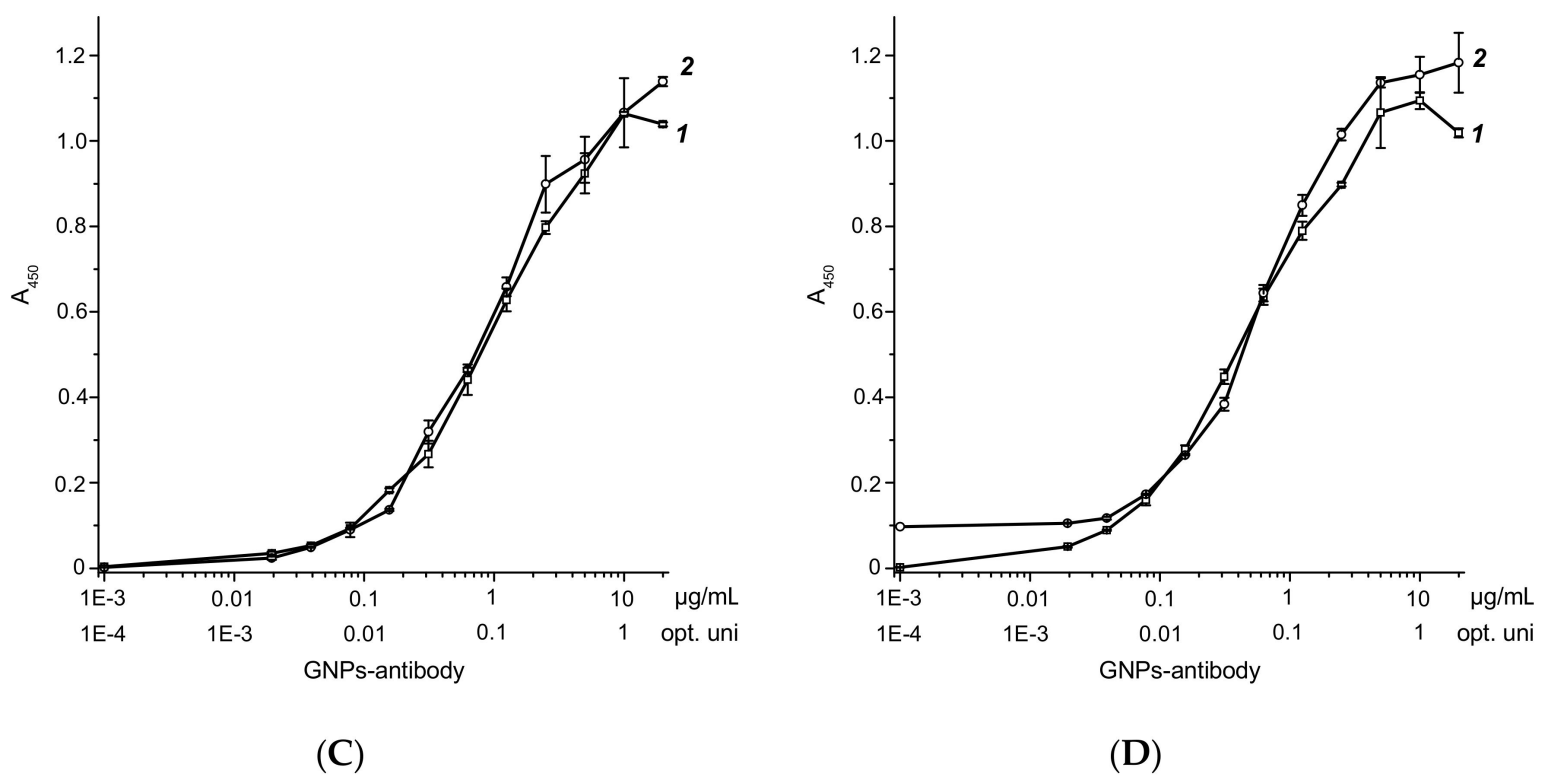
Functional characterization of the conjugate GNPs–antibody. Dependences of specific bindings of anti-GLRaV-3 antibody (**A**,**B**) and GNPs–antibody (**C**,**D**) to the goat anti-mouse immunoglobulin (GAMI; **A**,**C**) and the goat anti-rabbit immunoglobulin (GARI; **B**,**D**) on the antibody concentration. The curves correspond to immunoperoxidase conjugates of anti-mouse (1) and anti-rabbit (2) antibodies. The measurements were made in triplicate.

Due to the given complex composition, it was important to confirm that the conjugate also contains these two types of antibodies, which makes it possible to preserve the selectivity and affinity of the conjugate when interacting with viruses. For this purpose, the testing of the functional activity of the native anti-GLRaV-3 antibodies and their conjugates with GNPs was implemented. The experiment was conducted in a sandwich type of a microplate ELISA formation of triple complexes: (anti-species antibodies)–(anti-GLRaV-3 antibodies (native or conjugated with GNPs))–(enzyme-labeled anti-species antibodies). The results obtained in this experiment are presented in Figure 4. As you can see, both rabbit and mouse antibodies are detected in the conjugates, and the degrees of binding for native antibodies and conjugates (dilutions for the same antibody content) during the formation of sandwich complexes are comparable.

### 2.2. Preparation of Test Strips

In the prepared test strips, the diameter of GNPs was varied with the corresponding changes in the load of antibodies per one nanoparticle. Using three synthesized GNPs–antibody conjugates, three variants of immunochromatographic test systems were prepared. Concentrations of immunoreagents and modes of their application onto the working and fiberglass membranes were chosen based on data from our previous studies [42,43,44].

A mixture of the goat anti-mouse immunoglobulin (GAMI) and the goat anti-rabbit immunoglobulin (GARI) solutions was used to form the CZ, and the mixture of MAb and PAb specific to GLRaV-3 was used to form the TZ. Reagents were applied to the CZ and TZ of the working membrane from volumes of 2.0 µL per 1 cm width of the working membrane, which ensured the sorption uniformity and, accordingly, the minimum variation between test strips. 

GNPs–antibody conjugates were applied from a solution with A_520_ = 6.0 from volumes of 16.0 μL per 1 cm width of fiberglass membrane. This ensured the formation of intensely stained zones during the analysis in combination with the complete removal of the conjugates from the starting zone and the absence of non-specific staining of the working membrane.

The formation of the multimembrane composite was carried out under conditions chosen during our previous studies [42,43,44] and in accordance with generally accepted practice [45]. After applying the reagents, the membranes were dried for at least 20 h at 18–20 °C and successively fixed on a polystyrene substrate so that the edges of the membranes overlapped by 1–2 mm. The resulting composites were cut into test strips 3.5 mm wide.

### 2.3. Preparation of Pooled Positive Sample of Grape Leaf Extracts

67 samples were selected in the vineyards of the Crimea and the Krasnodar regions with symptoms resembling GLRaV-3. The samples were analyzed by the PCR and ELISA system Bioreba. Six samples of grape leaf extracts with the highest GLRaV-3 titers (NN 563, 565, 594, 596, 623, 626) were pooled in equal volumes and used as a pooled positive sample.

### 2.4. Characterization of the Developed Test System

**Figure 5:**
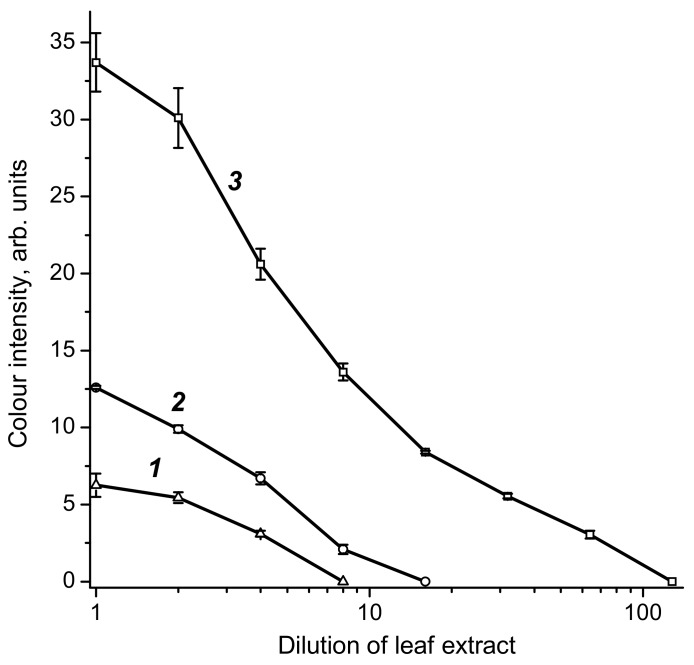
Plots of the color intensity vs. the dilution of pooled infected extract. The curves correspond to GNPs–antibody conjugates with GNPs diameters of 18.5 ± 3.3 nm (1), 28.3 ± 3.3 nm (2), and 51.0 ± 7.9 nm (3). The measurements were made in triplicate.

Using three variants of test systems differing in GNPs diameter, GLRaV-3 was detected in a pooled sample of grape leaf extract. The obtained results are given in Figure 5. It can be seen that when using a conjugate with GNPs with a diameter of 51 nm, the staining intensity of the test line in an undiluted pooled sample is 3–4 times higher and GLRaV-3 is revealed until the point where the sample is diluted 64 times—i.e., 4 times more than when using GNPs with a diameter of 28.3 nm, and 8 times more than when using GNPs with a diameter of 18.5 nm.

These experiments identify GNPs with a diameter of 51 nm as optimal for use in the developed test system. This label allowed for reliable detection in a wide range of GLRaV-3 dilutions, i.e., with minimal detectable concentration. The binding of the conjugate in the test zone did not prevent the interaction of the unreacted conjugate with anti-species antibodies in the control zone and the quality control of the test system according to the intensity of its staining.

### 2.5. Approbation of the Developed Test System

**Figure 6:**
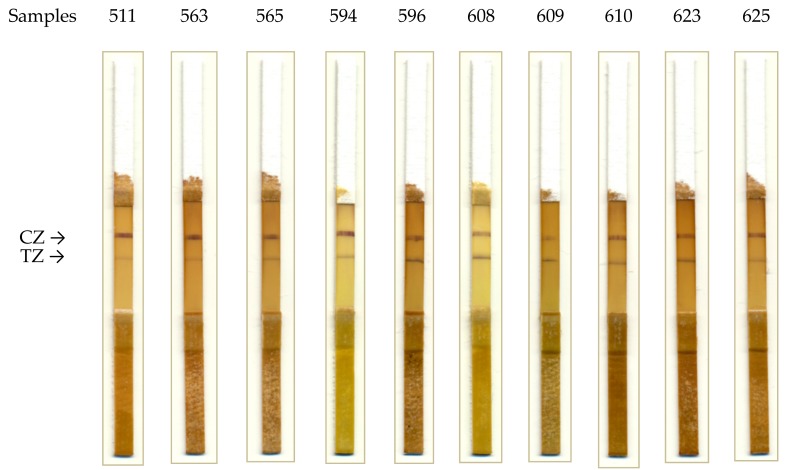
Results of ICA detection for GLRaV-3 in grape leaf extracts: test strips after analysis (CZ, control zone; TZ, test zone).

Leaves with visual symptoms of GLRaV-3 (n = 14), grapevine leafroll-associated virus 1 (GLRaV-1; n = 15), grapevine virus A (GVA; n = 15), and grapevine fanleaf virus (GFLV; n = 10), and without visual symptoms of infection (n = 13) were collected in 2017–2018 in the vineyards of the Crimea and the Krasnodar regions and were used to test the developed ICA. The presence or absence of viruses was confirmed using PCR and the ELISA test system Bioreba. Figure 6 shows the test strips after analyzing positive samples.

When analyzing samples containing GLRaV-1, GVA, and GFLV, there was no staining in the test zone of the test strips, which confirms the specificity of the test system.

The total test results are summarized in Table 1. As can be seen, when using ELISA as a reference method, the developed ICA is characterized by a sensitivity of 100% and a specificity of 92%. If PCR is considered as a reference method, then the sensitivity of ICA is 93%, and the specificity is 92%. Note that the detection of GLRaV-3 viruses is significantly influenced by the diversity of their strains [5], which changes both the genotype and antigenic properties. This may cause differences in the detection of the virus by ICA and ELISA, since they were implemented using different antibody preparations that were acquired against different viral strains as antigens. Nevertheless, the achieved levels of sensitivity and specificity are quite high for screening tests and confirm the promise of the developed ICA as the primary out-of-laboratory characterization of this viral grape infection.

## 3. Discussion

The developed assay provides a significant improvement in GLRaV-3 detection. Currently, commercially available immunotechniques for GLRaV-3 are only available via ELISA kits (Bioreba, Reinach, Switzerland, www.bioreba.ch; Creative Diagnostics, New York, NY, USA, www.creative-diagnostics.com; Loewe Biochemica, Sauerlach, Germany, www.loewe-info.com; Sediag, Bretenière, France, www.sediag.com). These assays may be implemented only with the use of special equipment such as a microplate washer and reader, and require stages under controlled heating (37 °C) and several prolonged incubations (from 1–2 h to overnight incubation). Thus, these assays may be realized only at some centralized laboratories with significant delay in obtaining diagnostic results. The same limitations are stored for ELISA protocols described in research publications [45]. The existing ICA for GFLV [32] requires duration of 30 min and is not supported by a line of tests for other grape wine viruses, including GLRaV-3.

The assay described in this paper is implemented in one stage, without the use of any additional reactants and devices. The testing results can be obtained in 10 min and detected visually. Thus, the presented study demonstrates the efficiency of immunochromatographic technology for rapid non-laborious on-site control of grape wine diseases. The realized approach is also transferrable for the detection of other grape wine pathogens.

## 4. Materials and Methods

### 4.1. Reagents

The present study used a mixture of MAb and PAb specific to GLRaV-3 (Bioreba, Reinach, Switzerland); GAMI and GARI (Arista Biologicals, Allentown, PA, USA); streptavidin–horseradish peroxidase conjugate, 3,3’,5,5’-tetramethylbenzidine dihydrochloride (TMB), polyvinylpyrrolidon K25 (PVP) with m.v. ~24 kDa, polyethylenglycol (PEG) with m.w. ~6 kDa, Tris, sucrose, Triton X-100, sodium citrate, chloroauric acid, sodium azide, agarose (Sigma-Aldrich, St. Louis, MO, USA), dimethyl sulfoxide (DMSO), Tween 20 (MP Biomedicals, Santa Ana, CA, USA), and bovine serum albumin (BSA) (Biowest, Nuaillé, France). All auxiliary reagents (acids, alkalis, salts, and organic solvents) were of analytical or chemical purity grade.

Solutions for syntheses of the GNPs and their conjugates with antibodies were prepared using deionized water, the resistivity of which at 25 °C was no less than 18.2 MΩ·cm (Simplicity, Millipore, Burlington, MS, USA).

ELISA was conducted using 96-well transparent microtitration plates (Costar 9018, Corning Costar, New York, NY, USA). To manufacture test strips, the following membranes were used: a nitrocellulose (NC) membrane grade CNPC with a pore size of 15 μm attached to a solid support, a conjugate release matrix PT-R7, a separation membrane GFB-R4, and an absorption membrane AP045 (all membranes are from Advanced Microdevices, Ambala Cantt, India).

### 4.2. Synthesis of GNPs

To obtain GNPs, the Frens method [46] was used with modifications described in [34]. First, 1.0 mL of 1.0% chloroauric acid solution was added to 98.25, 97.5, or 95.0 mL of deionized water and brought to a boil. Then, 0.75, 1.5, or 4.0 mL of 1.0% sodium citrate was added, respectively, while stirring. The mixtures were boiled (25 min), then cooled and stored at 4–6 °C. The spectra of the GNPs preparations were recorded with a Biochrom Libra S60 spectrophotometer (Biochrom, Cambridge, UK). 

### 4.3. Transmission Electron Microscopy

GNPs were applied to grids (300-mesh, Pelco International, Redding, CA, USA) coated with a poly (vinyl formal) film and registered by a JEM CX-100 electron microscope (JEOL, Tokyo, Japan). The obtained images were processed with the Image Tool program (University of Texas Health Science Centre at San Antonio, San Antonio, TX, USA).

### 4.4. Obtaining Flocculation Curves

Flocculation curves were obtained by the method described by Hermanson [39]. The antibodies were firstly dialyzed against a 1000-fold excess of 0.01 M Tris-HCl buffer, pH 9.0, at 4 °C for 2 h. Then, 0.1 M potassium carbonate was added to the GNPs solutions (A_520_ = 1.0) to reach pH 9.0. Next, 1.0 mL of the GNPs solutions (A_520_ = 1.0) was added to 0.1 mL of the antibody solutions (concentrations ranged from 5 μg/mL to 250 μg/mL), which were then mixed and incubated for 10 min at 20–22 °C. Then, 0.1 mL of 10% sodium chloride was added and stirred, and the A_580_ was measured after 10 min. The dependence of the A_580_ from the antibody concentration was used to choose the ratios of antibodies to GNPs during the synthesis of their conjugates.

### 4.5. Synthesis of Gnps–Antibody Conjugates

GNPs–antibody conjugates were prepared according to an established protocol [34,40]. GNPs solutions (the mean diameters 18.5 ± 3.3 nm, 28.3 ± 3.3 nm, and 51.0 ± 7.9 nm; A_520_ = 1.0; pH 9.0) were added to a mixture of MAb and PAb specific to GLRaV-3 in total concentrations of 5 μg/mL, 7 μg/mL, and 10 μg/mL, respectively. The mixtures were incubated for 45 min at 20–22 °C under stirring, after which an aqueous BSA solution was added (its final concentration was 0.25%). GNPs with immobilized antibodies were separated from non-reacted antibodies by centrifugation at 10,000 *g*, 8000 *g*, and 6000 *g* for 30 min. After the supernatant was removed, the residue was resuspended in 0.02 M Tris-HCl buffer, pH 7.6, containing BSA (1.0%), sucrose (1.0%), and Tween 20 (0.25%) (Tris-BSTA). Sodium azide (0.1%) was also added for prolonged storage. The preparations were stored at 4–6 °C.

### 4.6. Functional Characterization of The Conjugate Gnps–Antibody

The antibody content in the GNPs–antibody conjugates was determined by ELISA. The GAMI or GARI were coated by adding 50 μL of 1.0 μg/mL in 50 mM potassium phosphate buffer, pH 7.4, containing 0.1 M of sodium chloride (PBS) to each plate well and incubating at 4 °C overnight. The microplate wells were washed 4 times using PBS with 0.05% Triton X-100 (PBST). Then, for calibration curves, 50 μL of a mixture of MAb and PAb specific to GLRaV-3 (from 2 ng/mL to 2000 ng/mL) in PBST were added into the wells. For conjugate characterization, 50 μL of the GNP–antibody conjugate (from 0.002 to 2 optical units) diluted in PBST was added into the wells. The microplate was incubated for 1 h at 37 °C and then washed 4 times with PBST. At the next step, 50 μL of either anti-mouse or anti-rabbit immunoperoxidase conjugate (1:3000 dilution in PBST) was added into the wells, and this mixture was incubated for 1 h at 37 °C.

After washing, the peroxidase activity was determined. The substrate (50 μL of 0.4 mM TMB solution in 40 mM citrate buffer, pH 4.0, with 3 mM H_2_O_2_) was added to each well. After 15-min incubation at room temperature, 1 M H_2_SO_4_ (25 μL) was added to terminate the reaction and A_450_ was measured using a microplate photometer Zenyth 3100 (Anthos Labtec Instruments, Salzburg, Austria). The mono- and polyclonal antibodies contents in the GNP–antibody conjugates were calculated using the calibration curves obtained for free MAb and PAb as standards.

### 4.7. Preparation of Test Strips

An automatic dispenser (IsoFlow, Imagene Technology, Hanover, NH, USA) was used to apply the reagents to the membranes. A mixture of GAMI and GARI antibodies (at 0.75 mg/mL of each antibody in PBS, containing BSA (0.25%), sucrose (0.25%), and sodium azide (0.1%), PBS-BSA) and a mixture of MAb and PAb specific to GLRaV-3 (at 0.5 mg/mL of each antibody in PBS-BSA) were used to form the CZ and TZ on the NC membrane. 2.0 μL of solutions were applied per cm of CNPC membrane width. The GNPs–antibody conjugates were immobilized on a membrane in a dilution corresponding to A_520_ = 6.0 in Tris-BSTA. 16.0 μL of the solution was applied per cm of the PT-R7 membrane width. After the immunoreagents application, the CNPC and PT-R7 membranes were dried in air at 20–22 °C for at least 2 h. Then, these two membranes, the separation membrane GFB-R4 and absorption membrane AP045, were used to assemble sheets of multi-membrane composites. Next, an automatic guillotine cutter (Index Cutter-1, A-Point Technologies, Gibbstown, NJ, USA) was used to cut those sheets into strips of 3.5 mm width.

### 4.8. Preparation of Grapevine Leaves Extracts

Grapevine leaves with visual symptoms of defeat by GLRaV-3 (n = 14), GLRaV-1 (n = 15), GVA (n = 15), GFLV (n = 10), and without symptoms of infections (n = 13) were harvested in vineyards of the Crimea and Krasnodar regions.

Extracts were prepared by grinding a leaf sample with a mass of 200 ± 20 mg in 2 mL of extraction solution (0.2 M Tris-HCl buffer, pH 8.2, containing 0.8% sodium chloride, 2.0% PVP, 1.0% PEG, 0.05% Tween 20, and 0.02% sodium azide) for 1 min. Thus, the leaf/buffer weight ratio was approximately equal to 1:10.

The pooled infected leaf extract was prepared by mixing equal volumes of six extracts of positive (according to ELISA and PCR data) GLRaV-3 samples.

### 4.9. Lateral Flow Immunoassay

Assaying was performed at room temperature. The test strip was submerged in the sample vertically for 1.5 min, then it was removed and placed horizontally. The result was checked within 10 min of beginning the assay visually and by Canon CanoScan 9000F scanner (Canon, Tokyo, Japan). The obtained images were analyzed with a TotalLab TL120 software (Nonlinear Dynamics, Newcastle upon Tyne, UK) as described previously [43].

### 4.10. Reference ELISA Assay

For the reference assays of GLRaV-3 in leaf extracts, «Grapevine leafroll-associated virus 3 (GLRaV-3) DAS-ELISA» kits (Bioreba, Reinach, Switzerland) were used according to the manufacturer’s instruction.

### 4.11. PCR Analysis

From selected leaf samples, total RNA was isolated [47] and its quality was checked using electrophoresis. Reverse transcription (RT) was performed according to the protocol of the reagents manufacturer, Thermo Scientific. The quality of cDNA was verified by PCR with primers for the 18S rRNA gene [48]. For the detection of GLRaV-3, PCR was performed with primers on the envelope protein gene 547F 5’-ATTAACTTGACGGATGGCACGC-3’ and H229 5’-ATAAGCATTCGGGATGGACC-3’ [49]. RT-PCR products were separated in 1% agarose gel.

### 4.12. Analysis of Approbation Data for the Developed Test Strips

In accordance with standard statistical procedures [50], the test strips were applied to characterize positive leaf extracts (A samples) with the presence of GLRaV-3 confirmed by a chosen reference technique (either ELISA or PCR, see Section 2 and Section 3), and to characterize negative leaf extracts (B samples) with the absence of GLRaV-3 confirmed by the same reference technique.

The diagnostic sensitivity of the tests was calculated as A1/A, where A1 was the quantity of positive samples which the test confirmed the presence of the virus. The diagnostic specificity of the tests was calculated as B1/B, where B1 was the quantity of negative samples which the test confirmed the absence of the virus.

## Figures and Tables

**Table 1:** Results of ICA, ELISA, and PCR of GLRaV-3 in leaf extracts.

ICA Testing	Confirmation by ELISA	Confirmation by PCR
Positive—14	Positive—14 Negative—0	Positive—13 Negative—1
Negative—53	Positive—4 Negative—49	Positive—4 Negative—49

## Supplementary Materials

The following are available online at http://www.mdpi.com/2079-6374/8/4/111/s1, Figure S1: Transmission electron microscopy of GNPs: Images of GNPs (A,C,E) and histograms (B,D,F) of GNPs diameters (n = 93–153). The mean diameters are 18.5 ± 3.3 nm (A,B), 28.3 ± 3.3 nm (C,D), and 51.0 ± 7.9 nm (E,F).
