# Supplementary material for: Lateral Flow Immunoassay for Rapid Detection of Grapevine Leafroll-Associated Virus
Source: Biosensors (Basel). 2018 Nov 15;8(4):111. doi: 10.3390/bios8040111 (PMC6315891; doi:10.3390/bios8040111)
Supplement: Supplementary file 1 [file biosensors-08-00111-s001.pdf]

Electronic Supplementary Material

Lateral flow immunoassay for rapid detection of grapevine leafroll-associated virus

Nadezhda A. Byzova[1], Svetlana V. Vinogradova[2], Elena V. Porotikova[2], Yuliana D. Terechova[2], Anatoly V. Zherdev[1], Boris B. Dzantiev[1,*]

[1]A.N. Bach Institute of Biochemistry, Research Center of Biotechnology of the Russian Academy of Sciences, Leninsky prospect 33, Moscow, 119071 Russia
[2]Institute of Bioengineering, Research Center of Biotechnology of the Russian Academy of Sciences, Leninsky prospect 33, Moscow, 119071 Russia
* Correspondence: dzantiev@inbi.ras.ru; Tel.: +7-495-954-3142

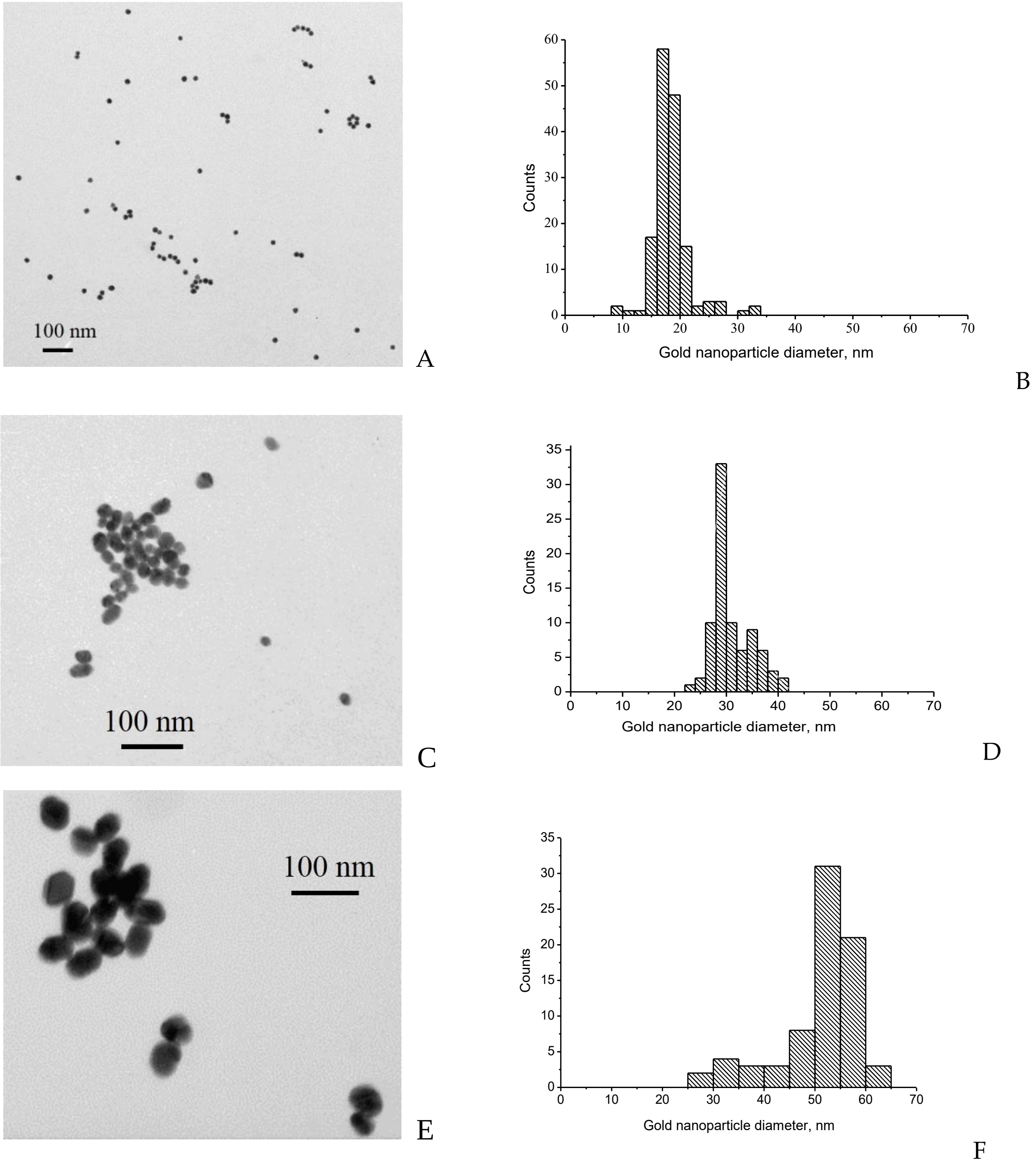

Figure S1. Transmission electron microscopy of GNPs: images of GNPs (A, C, E) and histograms (B, D, F) of GNPs diameters (n = 93-153). The mean diameters are 18.5 ± 3.3 nm (A, B), 28.3 ± 3.3 nm (C, D), and 51.0 ± 7.9 nm (E, F).
